# Erratum: Collective modes in multi-Weyl semimetals

**DOI:** 10.1038/srep46119

**Published:** 2017-06-21

**Authors:** Seongjin Ahn, E. H. Hwang, Hongki Min

Scientific Reports 6: Article number: 34023; 10.1038/srep34023 published online: 09 30 2016; updated: 06 21 2017.

This Article contains typographical errors, where all instances of \bm***k*** should read ***k***.

