# Supplementary material for: Collective modes in multi-Weyl semimetals
Source: Sci Rep. 2016 Sep 30;6:34023. doi: 10.1038/srep34023 (PMC5043176; doi:10.1038/srep34023)
Supplement: Supplementary Information [file srep34023-s1.pdf]

Collective modes in multi-Weyl semimetals

Seongjin Ahn[1], E. H. Hwang[2], and Hongki Min[1]
[1] *Department of Physics and Astronomy and Center for Theoretical Physics, Seoul National University, Seoul 08826, Korea and*
[2] *SKKU Advanced Institute of Nanotechnology and Department of Physics, Sungkyunkwan University, Suwon, 440-746, Korea*

Supplementary Information

A. Derivation of the long-wavelength polarization function for the isotropic model

In this section, we show the derivation of the analytical expressions for the zero-temperature polarization function and plasma frequencies in the $q \to 0$ limit. The polarization function for the isotropic model can be written as

$$\Pi(q,\omega) = g \sum_{s,s'=\pm} \Pi_{ss'}(q,\omega), \tag{1}$$

where

$$\Pi_{ss'}(q,\omega) = \int \frac{d^3k}{(2\pi)^3} \frac{f_{\boldsymbol{k},s} - f_{\boldsymbol{k}+\boldsymbol{q},s'}}{\hbar\omega + \Delta^{ss'}_{\boldsymbol{k},\boldsymbol{k}+\boldsymbol{q}} + i0^+} F^{ss'}_{\boldsymbol{k},\boldsymbol{k}+\boldsymbol{q}}. \tag{2}$$

Note that at zero temperature the Fermi distribution function is given by $f_{\boldsymbol{k},s} = \Theta\left(E_{\rm F} - E_{\boldsymbol{k},s}\right)$, where $\Theta(x)$ is the step function [$\Theta(x) = 0$ for $x < 0$ and $\Theta(x) = 1$ for $x \geq 0$]. We can break the polarization function into two parts due to intraband transitions and interband transitions, respectively: $\Pi(q,\omega) = \Pi_{\rm intra}(q,\omega) + \Pi_{\rm inter}(q,\omega)$, where $\Pi_{\rm intra}(q,\omega) = \Pi_{++}(q,\omega) + \Pi_{--}(q,\omega)$ and $\Pi_{\rm inter}(q,\omega) = \Pi_{+-}(q,\omega) + \Pi_{-+}(q,\omega)$. For convenience, we use the change of variable $\boldsymbol{k} \to -\boldsymbol{k} - \boldsymbol{q}$ and introduce dimensionless quantities $x = k/k_{\rm F}, y = q/k_{\rm F}, z = \hbar\omega/E_{\rm F}$ and $\Pi(q,\omega) = D(E_{\rm F})\widetilde{\Pi}(y,z)$, where $D(E_{\rm F})$ is the density of states at the Fermi energy given by $D(E_{\rm F}) = \frac{gk_0^3}{2\pi^2 N E_0}(E_{\rm F}/E_0)^{(3-N)/N}$.

Then we can rewrite $\widetilde{\Pi}_{\rm intra}(q,\omega)$ and $\widetilde{\Pi}_{\rm inter}(q,\omega)$ as

$$\widetilde{\Pi}_{\rm intra}(y,z) = \frac{N}{2}\int_0^1 x^2 dx \int_0^\pi \sin\theta d\theta \left(\frac{1}{z-\Delta_+} - \frac{1}{z+\Delta_+}\right) F_+ \tag{3}$$

and

$$\widetilde{\Pi}_{\rm inter}(y,z) = \frac{N}{2}\int_1^\infty x^2 dx \int_0^\pi \sin\theta d\theta \left(\frac{1}{z-\Delta_-} - \frac{1}{z+\Delta_-}\right) F_- \tag{4}$$

where $\Delta_\pm = (x^2 + y^2 + 2xy\cos\theta)^{\frac{N}{2}} \mp x^N$. In the $q \to 0$ limit $(z \pm \Delta_\pm)^{-1} \approx z^{-1}\left[1 \mp \frac{\Delta_\pm}{z} \pm \left(\frac{\Delta_\pm}{z}\right)^2 + \cdots\right]$ and $\Delta_\pm \approx (x^N \mp x^N) + N\cos\theta x^{N-1} y + \frac{1}{2}N\left\{1 + (N-2)\cos^2\theta\right\} x^{N-2} y^2 + \cdots$. In the presence of chirality the overlap factor is given by $F_\pm = \frac{1\pm\cos\theta'}{2} \approx \frac{1\pm 1}{2} \mp \frac{\sin^2\theta}{4x^2} y^2 + \cdots$, where $\cos\theta' = \frac{x+y\cos\theta}{\sqrt{x^2+y^2+2xy\cos\theta}} \approx 1 - \frac{\sin\theta^2}{2x^2} y^2$ whereas in the absence of chirality $F_+ = 1$ and $F_- = 0$. By putting all these equations into Eq. (3) and expanding up to the second order in y, we can obtain the long-wavelength polarization function. For the intraband part $\widetilde{\Pi}_{\rm intra}(y,z)$, the polarization functions for the chiral and non-chiral cases are the same up to the second order, given by

$$\widetilde{\Pi}_{\rm intra}(y,z) \approx \frac{N}{2}\int_0^1 x^2 dx \int_0^\pi \sin\theta d\theta \frac{2\Delta_+}{z^2} F_+ = \frac{N^2 y^2}{3z^2} + O(y^3). \tag{5}$$

For the interband part $\widetilde{\Pi}_{\rm inter}(y,z)$, note that there is no interband contribution for the non-chiral case due to the absence of interband transitions ($F_- = 0$). For the chiral case, $F_- \approx (\sin^2\theta/4x^2)y^2$ and when we expand the integrand only up to the second order, we can approximate $\Delta_- = 2x^N$, yielding

$$\widetilde{\Pi}_{\rm inter} y,z) = \frac{N}{2}\int_1^\infty x^2 dx \int_0^\pi \sin\theta d\theta \left(\frac{4x^N}{z^2-(2x^N)^2}\right)\frac{\sin^2\theta}{4x^2}y^2 + O(y^3) = \frac{2Ny^2}{3}\int_1^\infty dx \frac{x^N}{z^2-4x^{2N}} + O(y^3)$$
$$= -\frac{N\,{}_2F_1\left(1,\frac{N-1}{2N};\frac{3N-1}{2N};\frac{z^2}{4}\right)}{6(N-1)}y^2 + O(y^3) \text{ for } z<2, \tag{6}$$

where ${}_2F_1\left(a,b;c;z\right) = \frac{\Gamma(c)}{\Gamma(b)\Gamma(c-b)}\int_0^1 dt \frac{t^{b-1}(1-t)^{c-b-1}}{(1-tz)^a}$ is the hypergeometric function, which can be obtained after substituting $t = x^{-2N}$.

Thus the full polarizability in the long wavelength limit ($q \to 0$) is given by

$$\widetilde{\Pi}^{\rm nch}(y,z) = \frac{N^2y^2}{3z^2} \tag{7}$$

for the non-chiral case whereas

$$\widetilde{\Pi}^{\rm ch}(y,z) = \frac{N^2y^2}{3z^2} - \frac{N\,{}_2F_1\left(1,\frac{N-1}{2N};\frac{3N-1}{2N};\frac{z^2}{4}\right)}{6(N-1)}y^2 \text{ for } z<2 \tag{8}$$

for the chiral case. Note that the polarization function diverges when $N = 1$ because a linear band dispersion leads to the divergence of the polarization function at infinite cut-off[1]. By putting Eq. (7) and Eq. (8) into the dielectric function in Eq. (5) in the main text, we arrive at the analytic expressions for the leading-order long-wavelength plasma frequencies given in Eq. (8) and Eq. (9) in the main text.

B. Derivation of the long-wavelength polarization function in the anisotropic model

In this section, we show the derivation of the polarization function for the anisotropic case. Here we consider only the non-chiral case, where the overlap factor is given by $F_+ = 1$ and $F_- = 0$. Note that due to the anisotropic band structure it is no longer convenient to normalize the polarization function with the density of states as in the isotropic case. Thus for the anisotropic case we normalize the polarization function by $\widetilde{\Pi}(\widetilde{\boldsymbol{q}},\widetilde{\omega}) = \Pi(\boldsymbol{q},\omega)/\mathcal{C}$, where $\mathcal{C} = \frac{g}{(2\pi)^3}\frac{k_0^3}{E_0}$, $\widetilde{\boldsymbol{q}} = \frac{\boldsymbol{q}}{k_0}$ and $\widetilde{\omega} = \frac{\hbar\omega}{E_0}$. In the following, for brevity, the momentum and energy without tilde are considered to be normalized by k_0 and E_0, as in the main text.

We consider the following coordinate transformation:

$$\begin{aligned} k_x &= (r\sin\theta)^{\frac{1}{J}}\cos\phi, \\ k_y &= (r\sin\theta)^{\frac{1}{J}}\sin\phi, \\ k_z &= r\cos\theta. \end{aligned} \tag{9}$$

Note that with this coordinate transformation the energy dispersion becomes linear: $E_\pm(r) = \pm r$. The Jacobian corresponding to this transformation is given by $\mathcal{J}(r,\theta) = \frac{1}{J}\left(r\sin\theta\right)^{\frac{2-J}{J}} r$.

For the non-chiral case, there is no interband contribution, thus we consider only the intraband contribution. With a similar procedure as in the previous section, we can write the polarization function for the in-plane momentum $q_\|$ along the y-axis and out-of-plane momentum q_z along the z-axis as

$$\widetilde{\Pi}(q_{\|,z},\omega) = \int_0^{E_{\rm F}} dr \int_0^\pi d\theta \int_0^{2\pi} d\phi \mathcal{J}(r,\theta)\left(\frac{1}{\omega-\Delta_{\|,z}} - \frac{1}{\omega+\Delta_{\|,z}}\right) \tag{10}$$

where

$$\Delta_\| = E_{\boldsymbol{k}+\boldsymbol{q}_\|} - E_{\boldsymbol{k}} = \sqrt{r^2\cos^2\theta + \left[(r\sin\theta)^{\frac{2}{J}} + 2q_\|\sin\phi(r\sin\theta)^{\frac{1}{J}} + q_\|^2\right]^J} - r \tag{11}$$

and

$$\Delta_z = E_{\boldsymbol{k}+\boldsymbol{q}_z} - E_{\boldsymbol{k}} = \sqrt{r^2+q_z^2+2rq_z\cos\theta} - r. \tag{12}$$

In the $q \to 0$ limit, $(\omega \pm \Delta_{\parallel,z})^{-1} \approx \omega^{-1}[1 \mp \frac{\Delta_{\parallel,z}}{\omega} \pm \left(\frac{\Delta_{\parallel,z}}{\omega}\right)^2 + \cdots]$, $\Delta_z \approx q_z \cos\theta + \frac{\sin^2\theta}{2r} q_z^2 + \cdots$ and $\Delta_\parallel \approx A_1 q_\parallel + A_2 q_\parallel^2 + \cdots$ where $A_1 = J \sin\theta \sin\phi (r\sin\theta)^{\frac{J-1}{J}}$ and $A_2 = \frac{1}{4} J \sin\theta (r \sin\theta)^{\frac{J-2}{J}} \left[J \sin^2\phi(\cos 2\theta + 3) + 2\cos 2\phi\right]$.

By putting all these into Eq. (10), we get the following polarization functions:

$$
\begin{aligned}
\widetilde{\Pi}(q_\parallel, \omega) &\approx \int_0^{E_\mathrm{F}} dr \int_0^\pi d\theta \int_0^{2\pi} d\phi \mathcal{J}(r,\theta) \frac{2\Delta_\parallel}{\omega^2} \approx \frac{J\pi q_\parallel^2}{2\omega^2} \int_0^{E_\mathrm{F}} dr \int_0^\pi d\theta\, r \sin\theta (\cos 2\theta + 3) \\
&= \frac{4\pi J E_\mathrm{F}^2 q_\parallel^2}{3\omega^2}, \qquad (13) \\
\widetilde{\Pi}(q_z, \omega) &\approx \int_0^{E_\mathrm{F}} dr \int_0^\pi d\theta \int_0^{2\pi} d\phi \mathcal{J}(r,\theta) \frac{2\Delta_z}{\omega^2} \approx \frac{4\pi q_z^2}{J\omega^2} \int_0^{E_\mathrm{F}} dr \int_0^\pi d\theta\, (r\sin\theta)^{\frac{2-J}{J}}\, r \left(\frac{\sin^2\theta}{2r}\right) \\
&= \frac{\pi^{\frac{3}{2}} \Gamma\left(1 + \frac{1}{J}\right) E_\mathrm{F}^{\frac{2}{J}}}{\Gamma\left(\frac{3}{2} + \frac{1}{J}\right)} \frac{q_z^2}{\omega^2}. \qquad (14)
\end{aligned}
$$

Here we use the relation $\int_0^{\pi/2} d\theta \cos^m\theta \sin^n\theta = \frac{1}{2} B(\frac{m+1}{2}, \frac{n+1}{2})$ where $B(m,n) = \frac{\Gamma(m)\Gamma(n)}{\Gamma(m+n)}$ is the beta function. Note that linear terms in q_z vanish both in Eq. (13) and Eq. (14) due to the symmetry of the angular integrals. Combining Eq. (4) and the dielectric function in Eq. (5) in the main text, we arrive at the analytic expressions for the leading-order long-wavelength plasma frequencies given in Eq. (15) and Eq. (16) in the main text.

---

[1] J. Hofmann and S. Das Sarma, Phys. Rev. B **91**, 241108 (2015).
